# Physiological Data Analysis Framework for Pain Prediction in Physical Rehabilitation

**DOI:** 10.3390/s26134230

**Published:** 2026-07-03

**Authors:** Abdel Hiram Cital Duarte, Gilberto Borrego, Samuel González-López, Erica Cecilia Ruiz Ibarra

**Affiliations:** 1 Department of Electrical and Electronics, Instituto Tecnológico de Sonora, Ciudad Obregón 85130, Sonora, Mexico; abdel.cital@potros.itson.edu.mx (A.H.C.D.); erica.ruiz@potros.itson.edu.mx (E.C.R.I.); 2 Department of Computing and Design, Instituto Tecnológico de Sonora, Ciudad Obregón 85130, Sonora, Mexico; 3 Department of Mathematics, Universidad de Sonora, Hermosillo 83000, Sonora, Mexico; samuel.gonzalez@unison.mx

**Keywords:** pain prediction, physical rehabilitation, heart rate, heart rate variability, oxygen saturation, machine learning, telerehabilitation, biomedical signal processing

## Abstract

**Highlights:**

**What are the main findings?**

- Random Forest achieved 97.77% accuracy in detecting low-pain episodes using heart rate, HRV and SpO_2_ from only two low-cost wearable sensors during real rehabilitation sessions, demonstrating that simplified physiological monitoring can capture naturally occurring pain unlike prior laboratory-based studies.
- Preprocessing strategy significantly influenced model behavior: zero imputation favored low-pain detection (97.77%), interpolation improved moderate-pain balance (F1 = 0.708), and deletion provided the most balanced performance (76.64% accuracy), revealing no universal optimal configuration.

**What are the implications of the main findings?**

- Low-cost wearable-based pain monitoring can address patient underreporting in telerehabilitation by providing objective physiological evidence for timely therapy adjustments, improving adherence without increasing system complexity.
- Clinical objectives require tailored model-preprocessing combinations (RF-D0 for early detection of low pain and RF-Di/De for moderate pain), enabling lightweight orchestration strategies that improve robustness across diverse rehabilitation scenarios.

**Abstract:**

Predicting pain in physical rehabilitation is challenging due to subjectivity, patient variability, and self-report bias, especially in telerehabilitation. This study aims to determine whether machine-learning models based on heart rate (HR), heart rate variability (HRV), and peripheral oxygen saturation (SpO_2_) can reliably detect clinically meaningful pain during real rehabilitation sessions, including home-based settings where self-report is least reliable; we hypothesized that these low-cost, non-invasive markers carry sufficient information to flag low-to-moderate pain episodes without relying on self-report. We combined these markers with machine-learning models. These markers were selected for their association with autonomic pain responses and ease of measurement with only two low-cost, non-invasive sensors (a wearable band providing HR and HRV, and a fingertip oximeter providing SpO_2_) suitable for clinical and home-based rehabilitation. We evaluated linear regression (LR), random forest (RF), and artificial neural networks (ANNs) using data from 25 participants (aged 20–50) undergoing lower-limb rehabilitation. Signals acquired at 1 Hz were processed via temporal filtering, quality screening, and three missing-value strategies (interpolation, zero imputation, deletion) before normalization and training. LR showed limited predictive power. RF achieved 97.77% accuracy in detecting low-pain episodes, and balanced per-class performance under deletion (76.64%). ANN models contributed a more balanced three-class profile on interpolated data but remained sensitive to class imbalance. Given high-pain scarcity in supervised therapy and underreporting at home, reliable detection of low-to-moderate pain enables timely therapy adjustments. Unlike prior studies using experimentally induced pain, this work captured naturally occurring pain during real rehabilitation, making findings applicable to clinical and telerehabilitation contexts. Physiology-based models with low-cost sensors show promise for personalized rehabilitation, improving adherence and enabling proactive adjustments without added complexity.

## 1. Introduction

The International Association for the Study of Pain defines pain as an unpleasant sensory and emotional experience associated with actual or potential tissue damage [1]. Clinically, pain must be distinguished from nociception: the former is a conscious, context-dependent experience; the latter refers to neural activation by noxious stimuli [2]. In rehabilitation, musculoskeletal pain—frequent in the lower limbs—affects adherence and quality of life and modulates physiological responses such as heart rate variability (HRV) and oxygenation [3,4]. Traditional assessment relies on self-reports, which introduce variability and bias that hinder objective quantification across diverse clinical contexts [5]. This subjectivity is intrinsic to rehabilitation outcomes more broadly, where constructs such as pain perception and perceived exertion shape clinical decisions yet resist objective measurement [6].

These limitations are amplified in home-based rehabilitation. Patients may underreport symptoms due to minimization or perceived invalidation, which compromises timely treatment adjustments. Evidence links pain invalidation with underreporting, and systematic reviews of telerehabilitation highlight both benefits and the challenge of sustaining engagement and consistent reporting at home [7,8]. Within this pragmatic lens, the goal is not perfect scalar estimation but the dependable flagging of clinically meaningful episodes—especially moderate pain—that warrant intervention. As an illustration rather than a prerequisite, assistive systems such as MoveLeg [9] show how integrating physiological feedback into therapy can support this objective in both home and clinic.

**Figure 1:**
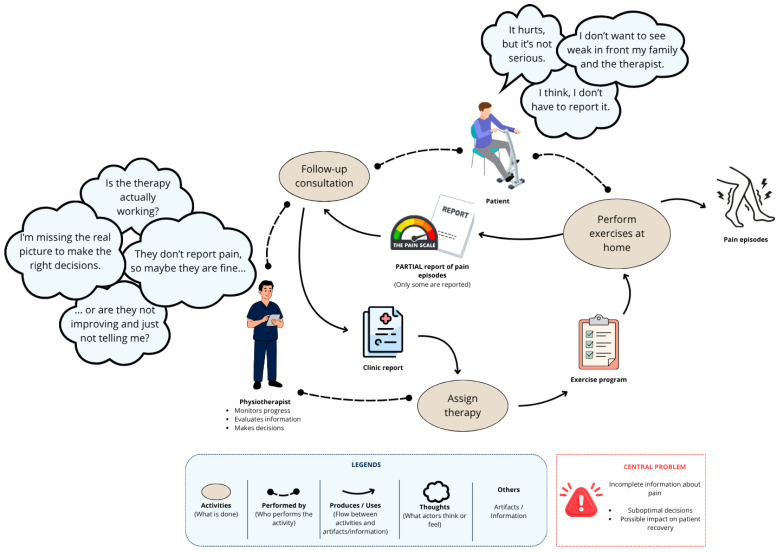
Rich picture of the current pain-reporting workflow in physical rehabilitation. During home-based exercise the patient experiences pain episodes, but only a partial subset reaches the follow-up consultation through self-report. The thought bubbles show common reasons for this attenuation (minimization, social self-presentation, and misjudged clinical relevance), and the therapist’s concerns illustrate the risk of making clinical decisions on incomplete evidence. The gap between actual and reported pain is the problem addressed in this study.

Furthermore, existing studies on physiological pain prediction have predominantly relied on experimentally induced pain under controlled laboratory conditions, limiting their clinical relevance; to our knowledge, no prior study has examined naturally occurring pain in real rehabilitation sessions, which represents the focus of the present work. Figure 1 illustrates this disconnect as a rich picture: the patient generates pain episodes during home-based exercise, yet only a partial subset reaches the follow-up consultation through self-report. The thought bubbles capture common reasons for this attenuation (minimization, social self-presentation, and misjudged clinical relevance) while the therapist’s concerns highlight the decision-making risk of acting on incomplete evidence. This mismatch between actual and reported pain is the problem this study addresses.

Building on these challenges, recent research has focused on methods based on physiological data. Biomarkers such as HRV, photoplethysmography (PPG), and other biomedical signals have shown potential correlations with pain perception, thus enabling a more automated approach to pain detection and management [10]. In this study, heart rate (HR) and its variability (HRV), together with oxygen saturation (SpO_2_), were selected as the physiological markers due to their accessibility, non-invasive measurement, and suitability for both clinical and home-based rehabilitation. HR and HRV are obtained from a single low-cost wearable band and SpO_2_ from a fingertip oximeter, keeping the setup to only two affordable sensors. HRV reflects autonomic nervous system activity and has been consistently associated with musculoskeletal pain responses, making it easily measurable with low-cost, widely available wearable sensors [2,3,11]. Similarly, SpO_2_ can be obtained with portable fingertip oximeters, which are affordable and user-friendly, facilitating integration into rehabilitation and telerehabilitation scenarios. Although its direct correlation with pain intensity remains less established, recent evidence highlights its complementary role when combined with other physiological markers in pain prediction frameworks [12,13].

The integration of artificial intelligence (AI) techniques has further facilitated the development of predictive models that can identify patterns in physiological data. These models are particularly relevant in rehabilitation, where assistive devices (e.g., robotic exoskeletons and automated orthotic systems) can utilize physiological feedback to enhance therapeutic outcomes [14,15,16,17]. The rise in telerehabilitation also opens new opportunities for incorporating AI-driven pain assessment into remote therapy, allowing continuous monitoring and personalized treatment adjustments. Nevertheless, applying these models clinically remains challenging due to the need for large, labeled datasets and the variability in pain perception among individuals [18]. Taken together, sensor acquisition, temporal signal processing, and machine learning constitute an integrated biomedical signal processing framework, which is the perspective adopted throughout this work.

Motivated by the gap and rationale outlined above, this study empirically compares three predictive models (linear regression, random forest, and artificial neural networks) to assess their effectiveness for pain prediction and to explore the clinical feasibility of low-cost, wearable-based physiological monitoring in naturally occurring rehabilitation pain. The proposed approach and its rationale are developed in the following sections, and the comparative results are reported in Section 4.

The rest of the article is structured as follows: Section 2 reviews related work and recent advances in pain prediction based on physiological data and AI. Section 3 details the methodology, including variables, instrumentation, data collection, and predictive modeling strategies. Section 4 presents the results. Section 5 discusses the findings and their clinical implications. Finally, Section 6 outlines the main conclusions and future research directions.

## 2. Related Work

A recent literature review [19] examined data analysis methods for predicting pain in physical rehabilitation, focusing on the combination of physiological signals with machine learning and artificial intelligence (AI) techniques. The review discussed various approaches using signals such as heart rate variability (HRV), electrocardiography (ECG), and photoplethysmography (PPG), and analyzed their effectiveness in predicting pain outcomes as well as their clinical implications. Screening proceeded through titles, abstracts, and full texts, retaining only studies that paired physiological monitoring with machine-learning techniques for pain assessment. The extracted data were analyzed to compare methodological approaches, signal-processing techniques, and the performance of predictive models. To avoid redundancy with the following subsections, we summarize only the high-level categories here and direct detailed discussion to Section 2.1, Section 2.2, Section 2.3 and Section 2.4. Building on these findings, recent studies have further expanded the scope of pain prediction by integrating physiological monitoring with emerging technologies such as Internet of Things (IoT)-based smart sensing [20], artificial intelligence frameworks for rehabilitation [14], and robotic coaching systems for physical therapy [15]. In parallel, dedicated reviews on automatic pain assessment have emphasized innovative methodologies and new perspectives [21], underscoring the rapid growth of this field in the last three years.

### 2.1. Pain Monitoring Methods

Researchers have explored several physiological signals as indicators for pain monitoring in physical rehabilitation. HRV, PPG, and ECG are recognized as valuable markers for pain assessment [5,18]. Studies indicate that HRV is a reliable marker for evaluating musculoskeletal pain [3]. Moreover, combining multiple signals can enhance the accuracy of pain assessment [12].

However, individual variability in these responses poses challenges. For example, although changes in HRV correlate with autonomic responses to pain, their relation to pain intensity remains inconclusive [22].

### 2.2. Pain Prediction Models

Advanced machine learning models have significantly improved pain prediction by extracting patterns from physiological data. Common algorithms include:

- Support Vector Machines (SVM): Widely used in pain state classification, with an average accuracy of 80% [5,11,23].
- Artificial Neural Networks (ANN): Achieved an accuracy of 74.19% in pain detection using electroencephalography (EEG) signals [24].
- Logistic Regression: Applied to combined PPG and HRV data, yielding a sensitivity of 60% and specificity of 72% [5].
- Random Forest (RF) and Linear Discriminant Analysis (LDA): Employed in various studies with performance dependent on the physiological signals analyzed [25,26].

### 2.3. Integration of Multiple Physiological Signals

Combining different physiological signals often improves pain prediction accuracy. For example:

- Models integrating PPG and HRV have reached an area under the curve (AUC) of 0.872 [5].
- The combination of heart rate (HR), breath rate (BR), and electromyography (EMG) achieved accuracies up to 83.3% [12].
- EEG-based pain assessment with neural networks reported an accuracy of 74.19% [24].
- Methods combining HRV, PPG, and galvanic skin response (GSR) demonstrated improved sensitivity and specificity compared to single-signal approaches [10,23].
- Additionally, recent integrative reviews on PPG provide updated insights into its diagnostic applications and potential for clinical implementation [13,27].

### 2.4. Clinical Applications and Challenges

Implementing AI models in clinical settings faces several obstacles:

- Data quality and availability: Collecting physiological data is costly and requires extensive preprocessing (e.g., normalization and labeling) to ensure reliable predictive modeling [5,22].
- Model interpretability: Although deep learning models can be highly accurate, their lack of explainability limits their use in clinical practice, where transparency is essential for medical decision-making [5,18].
- Interindividual variability: Differences in physiological responses complicate the development of universally applicable models, highlighting the need for adaptive techniques [10,12].

These factors underscore the importance of hybrid approaches that combine multiple signals with interpretable models, thus enhancing clinical applicability.

### 2.5. Traditional Models as an Alternative

Previous studies have evaluated traditional machine learning models (such as linear regression and random forest) as practical alternatives for pain prediction due to their advantages:

- Lower data requirements: These models can be trained with smaller datasets, making them more viable clinically.
- Higher interpretability: They facilitate the identification of key physiological patterns related to pain, aiding clinical decision-making [10].
- Clinical adaptability: Their ease of integration into existing medical monitoring systems improves real-world applicability [5].

Nonetheless, significant gaps remain, particularly regarding real-world rehabilitation contexts. Few studies have assessed pain prediction in real-world rehabilitation scenarios, limiting the clinical relevance of current approaches. Moreover, while traditional models reduce data needs and improve interpretability, their capacity to capture the complexity of pain perception is still uncertain. Taken together, these limitations of prior work—reliance on experimentally induced pain, multi-sensor or invasive setups, and the scarcity of evidence from real rehabilitation—constitute the rationale for the present study, which addresses them through a low-cost, two-sensor approach evaluated on naturally occurring pain.

RF has already been applied in pain prediction contexts. For example, a physiological signal-based method for pain intensity measurement using RF has been reported, achieving robust classification performance [28]. The present work extends this approach to real rehabilitation sessions rather than experimentally induced pain, highlighting ecological validity but also revealing challenges of low correlations.

Table 1 summarizes the methodological characteristics of prior pain prediction studies. As shown, all existing approaches relied on experimentally induced pain under controlled laboratory conditions, and most required multi-signal setups with specialized or invasive equipment. This consistent pattern across the literature reveals a gap: no prior study has assessed naturally occurring pain in real rehabilitation sessions using low-cost wearable sensors, which constitutes the primary motivation for the present work. The primary performance metric reported varies across studies: some report binary sensitivity/specificity, others multi-class accuracy (aggregate or per-subject median), and one reports AUC. This heterogeneity reflects differences in task formulation (binary vs. multi-class) and evaluation convention rather than true performance gaps, and precludes direct numerical comparison—a limitation we return to in Section 5. Because prior studies differ in task definition (binary vs. multi-class), signal modalities (single vs. multi-sensor), and pain source (experimentally induced vs. naturally occurring), the accuracy values in Table 1 are not directly comparable; they should be read as descriptive of each study’s setup rather than as a performance benchmark. The present work is among the few to evaluate naturally occurring pain in real rehabilitation sessions, and this methodological contrast—rather than raw numerical ranking—is what contextualizes its contribution.

### 2.6. Future Research Directions

The review identifies several areas for future research in pain prediction for rehabilitation:

- Integration of new physiological signals: Incorporation of new physiological signals could enhance model accuracy [12].
- Comprehensive multi-signal integration: More extensive combinations of signals are needed to boost predictive accuracy and clinical relevance [28].
- Application of advanced machine learning models: Sophisticated algorithms (e.g., deep learning) may further improve pain prediction in clinical contexts [18].
- Validation in real-world clinical environments: Testing model performance in actual rehabilitation settings is necessary for practical applicability [22].
- Larger sample sizes: Expanding datasets is crucial to generalize model findings [18,29].
- In addition, a 2022 scoping review explicitly addressed the role of AI and ML in pain prediction, identifying both methodological opportunities and current limitations [30]. Incorporating such findings strengthens the call for validation in real-world rehabilitation contexts and highlights the urgency of bridging experimental and clinical scenarios.

Implementing these strategies could substantially improve the development of precise and accessible pain monitoring tools for physical rehabilitation.

## 3. Methodology

This study aims to develop and evaluate predictive models for pain assessment in physical rehabilitation by analyzing physiological signals such as heart rate variability (HRV) and oxygen saturation. It seeks to identify patterns in these signals that correlate with pain perception, thus facilitating automated pain monitoring in clinical settings.

### 3.1. Participants

A total of 25 participants (14 men and 11 women) between 20 and 50 years old were recruited for this study. All participants presented musculoskeletal conditions localized in the lower body that required physical rehabilitation. Inclusion criteria required that participants were actively undergoing rehabilitation for conditions such as muscle strain, ligament injuries, or joint recovery processes, and that they were able to perform basic therapeutic exercises with supervision. Exclusion criteria were the presence of neurological disorders, cardiovascular diseases, or any condition that could compromise the accuracy of physiological signal measurement. All participants were informed about the objectives and procedures of the study and provided written consent in accordance with ethical regulations [31,32].

### 3.2. Variables

The study focuses on the following key variables:

- Self-reported pain intensity: Pain intensity was measured using the validated Numerical Rating Scale (NRS) [33], a continuous scale from 0 (no pain) to 10 (maximum pain). For analysis, these values were grouped into three categories: low pain (0–3), moderate pain (4–6), and high pain (7–10).
- Heart rate (HR), measured in beats per minute (BPM).
- Heart rate variability (HRV).
- Oxygen saturation (SpO_2_).

### 3.3. Instrumentation (Materials)

The following equipment was used for data collection:

- Heart rate monitor: COOSPO HW807 (Shenzhen CooSpo Tech Co., Ltd., Shenzhen, China; https://www.coospo.com/es/products/hw807-brazalete-pulsometro-pulsador, accessed on 29 June 2026), a wearable arm strap for continuous HR and HRV monitoring.
- Oxygen saturation sensor: FS20F (Shenzhen Viatom Technology Co., Ltd., Shenzhen, China; https://es.getwellue.com/p%C3%A1ginas/ox%C3%ADmetro-de-dedo-bluetooth-fs20f, accessed on 29 June 2026), a Bluetooth-enabled fingertip pulse oximeter for real-time SpO_2_ tracking.
- Data collection software: Custom Python 3.12 scripts for real-time signal acquisition and processing.

**Figure 2:**
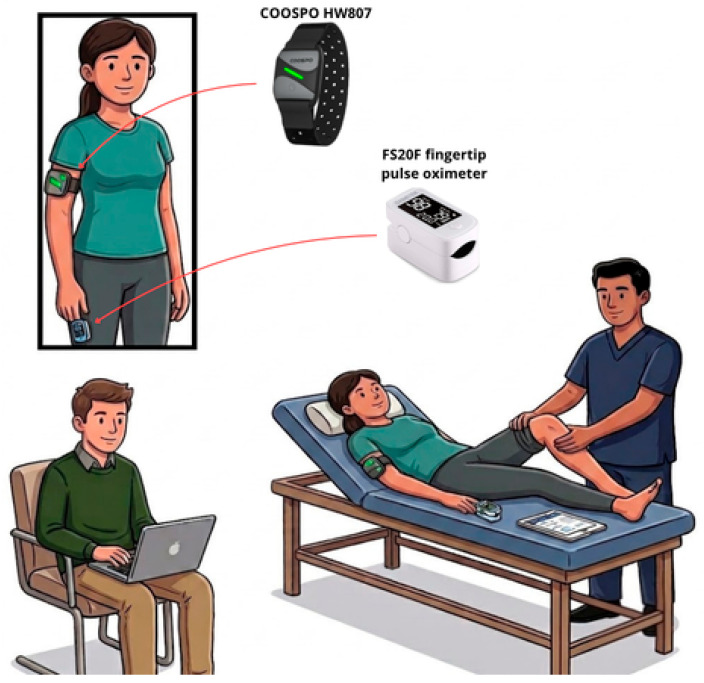
Conceptual scheme of the sensing setup. The framed inset (left) shows the placement of the two wearable devices on a participant, with callouts identifying each one: the COOSPO HW807 strap on the upper arm for heart rate and HRV, and the FS20F fingertip pulse oximeter for SpO_2_. The remaining panels show the devices worn during a therapist-assisted lower-limb rehabilitation session, with the physiological signals acquired in real time on a computer for later analysis.

The placement of both sensors on a participant is illustrated in Figure 2: the COOSPO HW807 strap is worn on the upper arm for continuous HR and HRV acquisition, and the FS20F pulse oximeter is clipped on a fingertip for SpO_2_ measurement, leaving the lower limbs free for the rehabilitation exercises.

### 3.4. Operation

This section describes the operational phase of the study, detailing how the experimental protocol was implemented in real rehabilitation sessions. It outlines the preparation of participants, the execution of therapy activities under sensor monitoring, and the procedures followed for data collection, validation, and model implementation. The purpose of this section is to provide a clear and structured overview of how physiological signals and self-reported pain data were captured, preprocessed, and integrated into predictive models for pain assessment.

#### 3.4.1. Preparation

The experimental setup was designed to capture data in a real clinical environment. Before each rehabilitation session, participants were equipped with the physiological sensors and instructed on the use of the Numerical Rating Scale (NRS). Proper sensor placement and device connectivity were verified prior to initiating the rehabilitation exercises. The protocol presented below was followed during all sessions.

1. Participant introduction: Participants received an overview of the study’s purpose and procedure.
2. Device setup: The heart rate band and pulse oximeter were placed on each participant for continuous monitoring.
3. Pain reporting instruction: Participants were instructed to report any pain verbally using a numerical scale (0–10), where 0 indicates no pain, and 10 indicates the highest pain.
4. Session execution: Participants engaged in rehabilitation activities while physiological monitoring was performed continuously throughout the session.
5. Session completion: Time was allocated for participants to ask questions or express concerns about the study.

The rehabilitation protocol focused on musculoskeletal conditions of the lower body. Pain was primarily movement-induced during stretching and strengthening exercises, and in some cases, therapy-induced by mechanical pressure. No experimental pain stimulation was applied; instead, naturally occurring discomfort during rehabilitation was recorded, thus reflecting clinically relevant pain episodes [3,33].

#### 3.4.2. Data Acquisition

Physiological signals were continuously acquired throughout each rehabilitation session while participants performed the prescribed therapeutic exercises. Recorded variables included heart rate (HR), heart rate variability (HRV), peripheral oxygen saturation (SpO_2_), and self-reported pain intensity using the Numerical Rating Scale (NRS). Pain scores were documented before, during, and after each session whenever participants reported pain or therapists observed visible discomfort.

Heart rate and HRV data were acquired from the COOSPO HW807 through a Bluetooth Low Energy (BLE) connection to a laptop using a custom Python application based on the Bleak library. Measurements were recorded at a fixed sampling interval of 1 s (1 Hz). Peripheral oxygen saturation (SpO_2_) was acquired from the FS20F pulse oximeter through its companion Android application, which recorded measurements at a nominal sampling rate of 1 Hz.

Each device generated an independent timestamped data stream (date and time, to the nearest second), which was exported after each rehabilitation session. The two streams were synchronized offline by aligning records with matching timestamps before being merged into a unified dataset for preprocessing and predictive analysis.

Missing measurements from the COOSPO, resulting from temporary Bluetooth communication interruptions, were recorded as NaN values by the acquisition software. For the FS20F, unreliable measurements caused by poor fingertip contact or motion artifacts typically appeared as physiologically implausible values outside the expected range and were therefore treated as missing. Missing observations from both sensors were subsequently handled using the interpolation (Di), zero-imputation (D0), and deletion (De) strategies described in Section 3.4.4.

#### 3.4.3. Execution

During rehabilitation sessions, participants wore the COOSPO HW807 and FS20F sensors to ensure continuous physiological monitoring throughout the rehabilitation exercises. Therapists supervised the rehabilitation activities, monitored participant safety, and ensured protocol compliance throughout the session. Each session lasted between 15 and 40 min, depending on the patient’s condition. Throughout the session, therapists monitored sensor placement and signal quality to minimize motion artifacts and poor sensor contact. Therapists supervised rehabilitation sessions, documented participants’ pain reports using the Numerical Rating Scale (NRS), and provided guidance when needed.

#### 3.4.4. Validation of Data

To ensure data quality and reliability, several preprocessing techniques were applied:

- Data normalization: Min-max scaling was used to standardize the range of physiological variables.
- Handling missing values: Three variants were implemented.
  - D0 (zero-imputed data), where missing fields were filled with the value 0 to preserve the length of the series.
  - Di (interpolated data), where linear interpolation was applied to estimate the missing values while maintaining temporal continuity.
  - De (deleted data), where records containing missing values were discarded, prioritizing fully informed observations.
- Noise filtering: Median and low-pass filters were applied to the 1 Hz time series of each physiological channel (BPM, RR intervals, SpO_2_) to suppress motion artifacts and sudden sensor dropouts, preserving the underlying autonomic trends relevant to pain assessment.
- Signal quality thresholds: Records were screened against established clinical norms; values outside the following ranges were flagged as anomalous and excluded: BPM outside 60–100 bpm, RR intervals outside 600–1000 ms, and SpO_2_ below 95% [34,35,36].
- Exploratory analysis: Correlation studies using both Pearson and Spearman coefficients (along with visual tools like histograms, scatter plots, and heat maps) were conducted to identify linear and non-linear relationships between physiological variables and reported pain. Pearson’s coefficient was used to quantify linear associations under approximate normality, whereas Spearman’s rank coefficient captured monotonic relationships without assuming normality or linearity; both were reported because the physiological variables did not consistently satisfy normality, and relying on a single measure could misrepresent the underlying associations.

These variants were consistently referenced throughout regression and classification experiments; for clarity across tables and figures, we use the abbreviations D0 = zero-imputed data, Di = interpolated data, and De = deleted (listwise) data, which are three alternative treatments applied to originally missing values.

#### 3.4.5. Predictive Models

To evaluate pain prediction in physical rehabilitation, three supervised learning approaches were implemented:

- Linear Regression: Employed as a baseline to evaluate trends between physiological variables and pain intensity. The model estimates pain intensity as a linear combination of the input features: ŷ = β_0_ + β_1_x_1_ + β_2_x_2_ + β_3_x_3_, where x_1_, x_2_, x_3_ correspond to BPM, HRV, and SpO_2_ respectively. Although simple and interpretable, its ability to model complex relationships is limited.
- Random Forest: An ensemble of decision trees that captures nonlinear relationships through bootstrap aggregation and random feature selection, combining predictions by majority vote (classification) or averaging (regression). It is robust to noise and provides feature importance estimates, making it suitable for physiological prediction.
- Artificial Neural Networks (ANNs): Used to probe more complex, non-linear relationships. Hidden layers employed rectified linear unit (ReLU) activation to introduce non-linearity and prevent vanishing gradients; output layers used Linear activation for regression tasks and Softmax for multi-class classification.

### 3.5. Model Validation

To ensure reproducibility and comparability across models, all experiments followed a standardized validation protocol:

- Data split: The dataset was partitioned at the participant level using an 80/20 split. All records belonging to a participant were assigned exclusively to either the training or testing subset, ensuring that no participant appeared in both datasets and preventing subject-level data leakage.
- Regression metrics: Mean Absolute Error (MAE), Root Mean Square Error (RMSE), and the coefficient of determination (R^2^) were computed. The pain labels were kept on their original 0–10 NRS and were not normalized; only the input features (BPM, HRV, SpO_2_) were min-max scaled. Consequently, MAE, RMSE, and R^2^ are reported on the original 0–10 pain scale, so MAE and RMSE are directly interpretable as errors in pain points.
- Classification metrics: Accuracy was complemented with confusion matrices to analyze true/false positives and negatives per pain class.
- Regularization and hyperparameter optimization: For ANN training, dropout and L2 penalties were applied. Hyperparameters were optimized using Keras Tuner with Random Search, exploring layer sizes (32–512 neurons, 2–4 layers), dropout rates (0.1–0.5), and L2 regularization coefficients. Early stopping and model checkpointing were used to avoid overfitting and retain the best-performing model. The best-performing architectures found through this search are summarized in Table 2.

## 4. Results

### 4.1. Data Distribution, Representative Patterns, and Correlations

Pain intensity was predominantly concentrated in the low and moderate ranges, with relatively few instances of high pain across participants. This distribution is consistent with the supervised nature of rehabilitation sessions.

**Figure 3:**
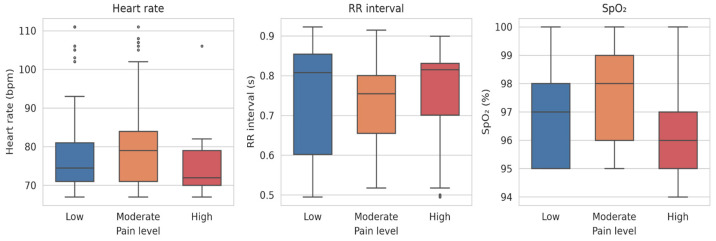
Boxplots of physiological parameters (heart rate, RR intervals, and peripheral oxygen saturation) stratified by pain level (low, moderate, high), computed from the 533 records with self-reported pain (low n = 170, moderate n = 238, high n = 125). Boxes show the interquartile range and median; whiskers extend to 1.5 × IQR, with outliers plotted individually.

Figure 3 shows how the three physiological markers distribute across the low, moderate, and high pain classes, computed from the 533 records in which pain was actually reported (low n = 170, moderate n = 238, high n = 125). The three classes overlap heavily in every marker, and none shows a monotonic shift as pain increases. Heart rate stays within a narrow band across classes (medians of 74.5, 79.0, and 72.0 bpm for low, moderate, and high), with the high-pain median in fact the lowest rather than the highest; this agrees with sessions conducted largely at rest, where limited physical activity keeps heart rate from tracking pain. RR intervals behave similarly (medians of 0.81, 0.76, and 0.82 s), with wide, overlapping interquartile ranges and no ordered trend. Oxygen saturation varies over a narrow clinical range (medians of 97%, 98%, and 96%): the high-pain class shows the lowest median, weakly consistent with the slight negative association reported below, but the moderate class is the highest, so no clear ordering holds.

**Figure 4:**
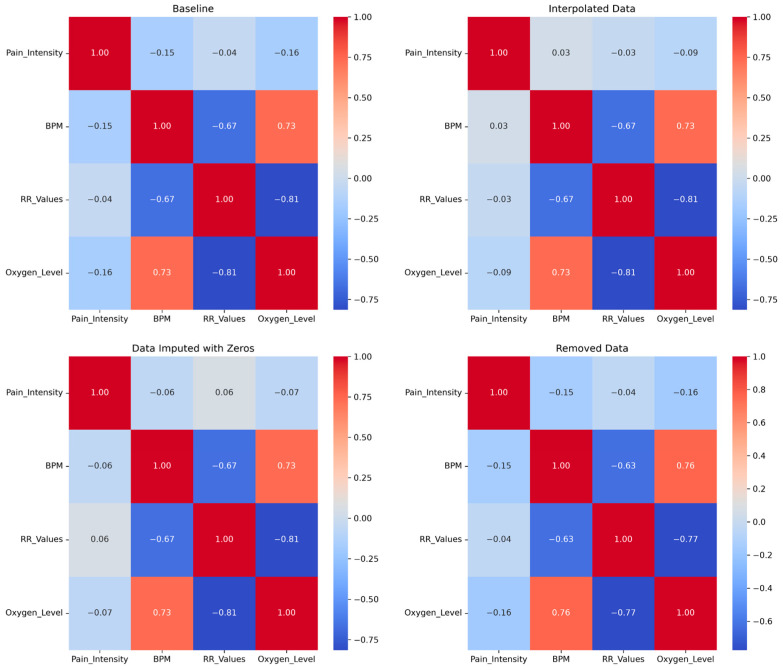
Pearson correlation heatmaps between physiological variables (BPM, RR intervals, oxygen saturation) and self-reported pain intensity, across four preprocessing variants: Baseline (raw), Interpolated (Di), Zero-imputed (D0), and Deleted (De). Values range from −1 (perfect negative) to +1 (perfect positive correlation).

The high-pain class is not only the smallest but also among the most dispersed and least consistent across markers, which helps explain why high pain is the hardest class to separate in the classification results reported later and is frequently confused with adjacent classes. Taken together, the boxplots reinforce, rather than contradict, the quantitative findings: the strong overlap and the absence of a monotonic trend agree with the weak correlations summarized in the heatmaps (Figure 4) and indicate that no single physiological variable, used on its own, reliably distinguishes pain levels. The limited class separation the models achieve emerges only when the three markers are combined, never from any single one, and the moderate and high classes remain the hardest to resolve, in line with the per-class results.

Representative patient data are presented in Table 3, illustrating physiological responses across different pain levels. These cases reveal heterogeneous patterns: some participants show increased BPM at moderate pain levels, while others exhibit irregular fluctuations or minimal variation. No consistent linear trend is observed across individuals, reinforcing the variability of physiological responses to pain.

Physiological variables, particularly heart rate and heart rate variability (HRV), exhibited substantial inter-individual variability. Correlation analysis between physiological signals and self-reported pain levels revealed generally weak associations. Both Pearson and Spearman coefficients yielded consistent results, confirming that these weak associations are stable across data variants rather than dependent on the choice of correlation measure. As illustrated in Figure 4 (heatmaps), heart rate and HRV show low correlation coefficients with pain, while oxygen saturation exhibits a slight negative correlation. These findings are numerically summarized in Table 4 and Table 5.

### 4.2. Regression Model Performance

The regression results are summarized in Table 4. Linear regression, used as a baseline, showed limited predictive capability, with a low coefficient of determination (R^2^) and relatively high error values (mean absolute error, MAE, and root mean square error, RMSE), indicating poor generalization across participants.

In contrast, Random Forest demonstrated improved performance, achieving lower MAE and higher R^2^ across preprocessing variants (D0, Di, De). However, performance varied depending on the treatment of missing data.

Artificial neural network (ANN) regression, trained on interpolated data (Di), achieved an R^2^ of approximately 0.23 and an RMSE of 1.49, reflecting modest predictive power in capturing the relationship between physiological variables and pain intensity.

### 4.3. Classification Performance: Random Forest

Classification results for Random Forest are presented in Table 5. To ensure comparability, classification results are reported using accuracy as the primary metric, while precision, recall, and F1-score are provided for class-level analysis. Overall, Random Forest achieved the highest performance among the evaluated models, particularly for low pain detection.

- Under D0 (zero imputation), the model reached an accuracy of 97.77%, with very high precision and recall for the low pain class. However, moderate and high pain were substantially underdetected.
- Under Di (interpolation), overall accuracy decreased (~60.65%), but class-level performance became more balanced. Notably, the moderate pain class achieved the highest F1-score (0.708), while low pain decreased (0.440).
- Under De (deletion of incomplete records), the model achieved an accuracy of 76.64%, providing the most balanced performance across classes, albeit with a reduced sample size.

These results indicate that classification behavior is strongly influenced by preprocessing strategy.

### 4.4. ANN Configuration Summary

ANN architectures and training configurations are summarized in Table 2, including layer structure, dropout rates, L2 regularization, and learning rates used for regression and classification tasks.

### 4.5. Classification Performance: Artificial Neural Networks

**Figure 5:**
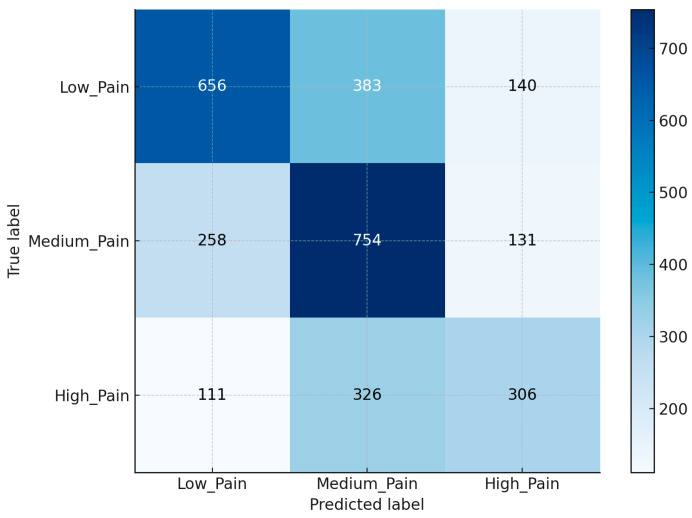
Confusion matrix for three-class ANN classification (interpolated data).

ANN classification results are also summarized in Table 5, with additional detail in Figure 5.

For three-class classification using interpolated data (Di), the ANN achieved an overall accuracy of 57.29%. The confusion matrix in Figure 5 highlights stronger discrimination for low and moderate pain, with systematic misclassification of high pain into adjacent classes.

In one-vs-rest configurations, class-wise accuracies were:

- Low vs. others: 70.96%
- Moderate vs. others: 64.67%
- High vs. others: 78.79%

A binary formulation (No pain vs. Pain) was also explored using zero-imputed data. However, the resulting classifier collapsed to the majority class under the prevailing class imbalance, labeling nearly all test instances as “No pain” and producing non-informative per-class precision, recall, and F1-score for the minority “Pain” class. Because aggregate accuracy in this regime is misleading—high by construction rather than by discriminative ability—the configuration is excluded from the comparative results in Table 5; its interpretation is addressed in Section 5.3.

## 5. Discussion

### 5.1. Interpretation of Findings

The results indicate that physiological responses to pain are inherently nonlinear and subject-dependent. The superior performance of Random Forest over linear regression (Table 4) reflects its ability to capture complex interactions between physiological variables, which are not adequately modeled by linear approaches.

Artificial neural network (ANN) models further explored nonlinear relationships, but their performance remained sensitive to data distribution and variability, as reflected in the classification results (Table 5 and Figure 5).

### 5.2. Impact of Preprocessing Strategies

Preprocessing had a substantial effect on model behavior. As shown in Table 5:

- D0 favored high accuracy for dominant classes (low pain) but introduced bias.
- Di improved temporal continuity, leading to better detection of moderate pain.
- De provided more balanced performance at the cost of reduced sample size.

These findings highlight a trade-off between data completeness, temporal consistency, and class balance.

### 5.3. Class Imbalance and Detection Asymmetry

A consistent pattern across models is the higher accuracy in detecting low pain compared to moderate and high pain (Table 5). This asymmetry is attributable to both class imbalance and greater variability in higher pain levels, which is already apparent in the data: in Figure 3, the high-pain class shows the widest and least consistent distributions across the three markers.

The confusion patterns observed in Figure 5 further support this, showing systematic misclassification of high pain into adjacent categories. This indicates that distinguishing between moderate and high pain remains a challenging task.

A concrete instance of this asymmetry was observed when the binary (No pain vs. Pain) ANN was trained on zero-imputed data: under the prevailing imbalance, the model converged on the majority class, producing a deceptively high aggregate accuracy (~96%) while yielding effectively no recall for pain events. Its per-class metrics are therefore not reported, and the configuration is excluded from the model-selection recommendations. This outcome reinforces the broader point of this subsection—that accuracy alone is an inadequate summary in imbalanced clinical settings, and that per-class recall must drive model selection when minority events carry the clinically relevant signal.

### 5.4. Clinical Implications

From a clinical perspective, the reliable detection of low and moderate pain is particularly relevant. In real rehabilitation settings, especially in telerehabilitation, patients often underreport low-intensity pain. Beyond pain intensity itself, subjective factors such as pain perception and perceived exertion are key parameters in the decision models physiotherapists use to tailor rehabilitation [6], reinforcing the value of objective physiological indicators that complement patient-reported measures.

In supervised rehabilitation, high-pain events are relatively rare due to clinical safeguards, whereas low-to-moderate pain episodes are more frequent and actionable for therapy adjustment.

The ability to detect these early signals—highlighted by the high performance of RF-D0 in low-pain detection and the balanced per-class profile of RF-De (Table 5)—enables:

- Early intervention and adjustment of therapy protocols.
- Prevention of pain escalation.
- Improved adherence to rehabilitation programs.

Thus, clinical utility lies not in perfect classification of all pain levels, but in consistent detection of actionable events.

**Figure 6:**
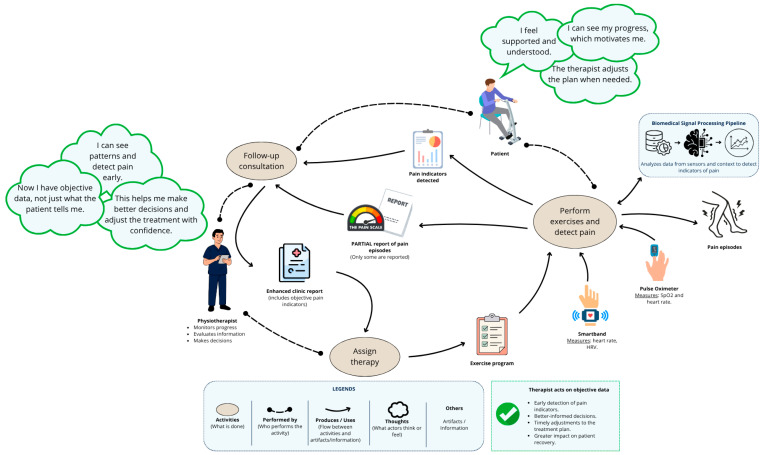
Proposed physiological monitoring framework integrating two low-cost wearable sensors (a smartband for heart rate and HRV, and a pulse oximeter for SpO_2_), a biomedical signal processing pipeline, and objective pain detection into the rehabilitation workflow.

Figure 6 restores the workflow of Figure 1 with the monitoring framework in place. Low-cost wearables (oximeter and wristband) now feed a biomedical signal processing pipeline that runs alongside the exercise activity, producing an objective stream of pain indicators that reaches the follow-up consultation in parallel with—not in place of—the patient’s partial self-report. The therapist therefore consults two complementary sources: subjective narrative and physiological evidence. The positive framing in the actors’ thoughts reflects the downstream effect: the patient feels supported because the system compensates for what is not verbalized, and the therapist gains confidence in acting on objective data—operationalized by the outcomes listed in the bottom-right callout (early detection, better-informed decisions, timely adjustments, greater impact on recovery).

Beyond the per-class metrics, the present framework was developed under an explicit deployment constraint: rehabilitation continues at home, where clinical-grade instrumentation is unavailable, costly, and impractical for routine self-application. The chosen sensing stack (a fingertip pulse oximeter and a consumer-grade wristband) was therefore not a compromise driven by equipment availability, but a design requirement aligned with the realities of community and home-based care, particularly in resource-constrained settings where high-end physiological monitoring is unattainable. Framing the sensor selection this way clarifies that the modest per-class performance reported in Section 4 should be read against the upper bound imposed by low-cost, single-modality wearables operating in unsupervised home conditions, rather than against laboratory studies using clinical-grade multi-sensor arrays. This deployment-aware framing (visualized in Figure 1 and Figure 6, which contrast the current self-report-only workflow with a system that surfaces objective pain indicators using the same accessible hardware) is what positions the contribution as ecologically valid: the goal is not maximal accuracy under controlled conditions, but actionable information under the conditions in which patients actually rehabilitate.

### 5.5. Comparison with Prior Work

As summarized in Table 1, most previous studies rely on experimentally induced pain and multi-signal setups. In contrast, the present work uses only heart rate (HR), heart rate variability (HRV), and peripheral oxygen saturation (SpO_2_), obtained from just two low-cost sensors, in real rehabilitation sessions.

Despite this simplified and more realistic setting, the proposed models achieve competitive performance, particularly for low pain detection and binary classification. This supports the feasibility of deploying low-cost, wearable-based monitoring systems in clinical practice.

Moreover, few studies in the literature have specifically combined HR, HRV, and SpO_2_ as the sole physiological markers for pain assessment in rehabilitation contexts. Most approaches incorporate multiple signals (e.g., EMG, GSR, ECG, PPG) or rely on more complex sensor arrays [5,12,23,28], which increase cost and deployment complexity.

The present work demonstrates that a minimal two-sensor setup can achieve competitive performance when applied to naturally occurring pain, supporting the feasibility of scalable, low-barrier monitoring solutions.

Direct quantitative comparison with prior work remains challenging due to differences in task definitions (binary vs. multi-class classification), signal modalities (single vs. multi-sensor setups), and pain sources (experimentally induced vs. naturally occurring). As noted in Table 1, accuracy metrics across studies reflect these methodological variations rather than strictly comparable performance benchmarks.

Nonetheless, the consistency of our findings with existing evidence—particularly the effectiveness of Random Forest for pain classification [28]—reinforces the validity of the proposed approach while highlighting its distinct contribution: ecological validity through real-world data collection.

### 5.6. Methodological Implications

Model-preprocessing combinations were selected based on alignment with clinical objectives (e.g., early detection vs. balanced classification) rather than exhaustive optimization.

The results suggest that no single model-preprocessing configuration is optimal for all tasks. Instead:

- RF-D0 is most effective for low-pain detection.
- RF-De yields the most balanced performance across low, moderate, and high pain, at the cost of a reduced effective sample size.
- RF-Di improves temporal continuity and favors moderate-pain discrimination.
- ANN-Di offers a complementary per-class profile in the three-class setup, and is a candidate for further refinement under class-balancing strategies.

This opens the possibility of combining models through lightweight ensemble strategies tailored to specific clinical objectives.

### 5.7. Practical Recommendations

Based on the observed results, different model-preprocessing combinations are recommended depending on the target application. RF-D0 is most effective for early detection of low pain due to its high accuracy on that class. RF-Di and RF-De provide more balanced performance for moderate pain, depending on whether temporal continuity or data completeness is prioritized, with RF-De additionally offering the most balanced profile across all three pain levels. ANN-Di is retained as a baseline for three-class classification, to be refined in future work with class-balancing strategies.

These findings suggest the feasibility of a lightweight orchestration strategy, in which different models are selectively applied depending on the operating context, improving overall system robustness without increasing computational complexity.

### 5.8. Limitations

Several limitations should be acknowledged. First, the sample size is relatively small (n = 25), and high-pain instances are underrepresented in the dataset. Additionally, correlations between individual physiological variables and pain are weak, and model performance is sensitive to preprocessing choices and data partitioning. For these same reasons, formal k-fold cross-validation and statistical model-comparison tests (e.g., Friedman or Wilcoxon) were not adopted as primary evidence: with only 25 participants and a near-absent high-pain class, cross-validation folds become unrepresentative (our exploratory cross-validation in the ANN regression setting yielded negative R^2^ values) so per-fold dispersion and significance tests computed on such unstable partitions would be misleading rather than informative. This instability is consistent with prior evidence that k-fold cross-validation yields biased, high-variance performance estimates under limited sample sizes [37,38] and is highly sensitive to the specific fold and training-set composition [39]; moreover, the standard remedy for this bias, nested cross-validation, requires still finer subdivision of the data than our 25-participant cohort, with a near-absent high-pain class, can sustain. We therefore report single held-out estimates and leave k-fold cross-validation with paired statistical testing to future work on larger cohorts.

The subject-level 80/20 partition adopted in this study preserves the independence between training and testing participants while providing sufficient data for model development. Nevertheless, alternative subject-level validation strategies, such as Leave-One-Subject-Out (LOSO) cross-validation, could provide complementary evidence regarding subject-level generalizability. Applying LOSO to the current cohort, however, would face specific instability challenges: with n = 25, each LOSO fold evaluates on a single held-out participant; given that high-pain instances are sparse (125 of 533 total records), many individual test subjects may contribute zero or very few high-pain observations, rendering per-class metrics undefined or highly unstable for those folds. Heterogeneous session durations (15–40 min) further produce unequal pain-record counts per participant, so fold-to-fold class distributions are non-uniform and aggregate LOSO estimates are difficult to interpret reliably. Additionally, the demographic range of the cohort (14 men and 11 women, aged 20–50) means that excluding a single participant can disproportionately remove a specific demographic profile from the evaluation, biasing the generalizability estimate. LOSO evaluation on a larger, demographically balanced cohort with sufficient representation of all pain classes therefore constitutes an important direction for future research.

Despite the limited sample size, the scale of this study is consistent with prior work in physiological pain prediction. As shown in Table 1, several studies report similarly small cohorts (e.g., n = 30 in [12]) or do not report sample size (NR), while many rely on controlled experimental conditions rather than real clinical environments.

Importantly, unlike most prior studies based on experimentally induced pain, the present work captures naturally occurring pain during real rehabilitation sessions. Although this introduces greater variability, it enhances ecological validity, which is critical for real-world and telerehabilitation applications. Therefore, while the sample size remains a limitation, it reflects the practical constraints of clinical data collection and aligns with the scale of comparable studies in the literature.

### 5.9. Future Directions

Future work should focus on:

- Addressing class imbalance through resampling or weighted loss functions
- Incorporating additional physiological signals
- Expanding the dataset to improve generalization
- Validating models in real-world telerehabilitation environments
- Exploring ensemble approaches to combine model strengths

## 6. Conclusions

This study evaluated the feasibility of using physiological signals such as heart rate (HR), heart rate variability (HRV), and oxygen saturation to predict pain levels in rehabilitation settings by comparing three predictive models: linear regression, random forest, and artificial neural networks (ANNs). Unlike many studies based on experimentally induced pain, this research used data from real rehabilitation sessions, thereby reflecting actual clinical conditions. Random forest achieved the strongest multiclass performance and high accuracy in detecting low pain, while ANN offered a more balanced per-class profile in the three-class setup but remained sensitive to data imbalance. In contrast, linear regression offered only limited predictive capabilities but served as a simple and interpretable baseline.

Despite these advantages, the random forest model struggled with intermediate pain levels, and both RF and ANN remained sensitive to data imbalance, underscoring the need for refined feature selection, class-balancing strategies, and larger datasets. Additionally, cross-validation experiments conducted during ANN regression exploration yielded negative R^2^ values, indicating sensitivity of model performance to the specific train/test partition. This highlights the need for k-fold cross-validation across all model types in future work to obtain more robust and generalizable performance estimates. Pain category thresholds were defined following standard Numerical Rating Scale (NRS) conventions (low: 0–3, moderate: 4–6, high: 7–10); however, exploratory experiments with alternative boundaries revealed sensitivity of classification results to threshold definitions, underscoring the importance of standardized pain categorization protocols. These challenges highlight the inherently variable nature of pain perception and the difficulty of developing universally reliable models.

The practical value of physiology-based detection does not lie in perfect accuracy, but in reliably recognizing episodes of moderate pain that patients may underreport or forget in home-based rehabilitation. Such reliable detection is essential for maintaining adherence and enabling timely protocol adjustments, aligning with evidence that self-reports often underestimate pain due to invalidation biases [7] and with telerehabilitation studies that highlight the need for improved monitoring in home environments [8]. Our findings support level-aware decisions: RF-D0 as an early detector for low pain, and RF-De/Di for moderate pain when completeness or continuity is prioritized. Given that high-pain events are rare in supervised practice, utility lies in promptly capturing low-to-moderate events to adjust protocols. A pragmatic next step is a lightweight ensemble to orchestrate these outputs, coupled with class-balance strategies and larger cohorts to improve sensitivity without increasing system cost.

Beyond model performance, a key contribution of this work is its ecological validity: by capturing naturally occurring pain during real rehabilitation sessions rather than experimentally induced stimuli, the findings reflect conditions that patients and therapists actually face. This is particularly relevant in telerehabilitation, where patients may underreport or forget low-intensity pain episodes between sessions. Crucially, the framework’s accessible sensing stack is a deliberate design choice rather than a methodological limitation: by relying on a fingertip pulse oximeter and a consumer-grade wristband, the pipeline preserves applicability in the home and community settings where rehabilitation actually unfolds, including resource-constrained contexts where higher-end instrumentation is not viable. It thus offers a practical path toward objective, continuous pain monitoring that complements therapist judgment without imposing additional burden on the patient.

## Figures and Tables

**Table 1:** Comparison of pain classification performance with prior literature.

Study	Model	Signals	n/Pain Source	Task	Best Metric	Key Observation
[5]	Logistic Regression	HRV + PPG	NR/Induced (heat)	Binary	Sens. 60%, Spec. 72%	Induced pain; similar signals to present work; lower sensitivity
[23]	SVM	EMG + ECG + SCL	90/Induced (heat)	Multi-class	>80% (individual)	Induced pain; multi-signal; high-cost equipment
[12]	ANN	HR + BR + GSR + EMG	30/Induced (thermal + electrical)	3-class	70.6% (83.3% median)	Induced pain; 4 signals; EMG required; not wearable-ready
[28]	RF/SVM/LDA	BVP + ECG + SCL	NR/Induced (electrical)	Multi-class	Not reported (robust classification)	Induced pain; multi-signal; RF + SVM combination
[24]	ANN	EEG	NR/Induced	Binary	74.19%	EEG-based; high cost; not wearable
[5]	Logistic Reg.	PPG + HRV	NR/Induced	Binary (AUC)	AUC 0.872	Best AUC reported with combined signals; induced pain

Note: NR = not reported. n = number of participants. Signals: HRV = heart rate variability; PPG = photoplethysmography; BVP = blood volume pulse; SCL = skin conductance level; EMG = electromyography; ECG = electrocardiogram; BR = breath rate; GSR = galvanic skin response; Metrics: Sens. = sensitivity; Spec. = specificity; AUC = area under the ROC curve; median = per-subject median.

**Table 2:** Artificial Neural Network (ANN) configurations for regression and classification on rehabilitation data.

Task	Dataset	Split	Layer Configuration	Dropout	L2 Regularization	Learning Rate
Regression	Interpolated data	80/20	[160, 64, 1]	[0.4, 0.1]	[0.001, 0.008]	0.0017
Three-class classification	Interpolated data	80/20	[256, 128, 160, 128, 32, 3]	[0.2, 0.4, 0.2, 0.3, 0.2]	[0.001, 0.0003, 0.009, 0.005, 0.0001]	0.0015
Three-class (one-vs-rest)	Interpolated data	80/20	[32, 1]	[0.2]	[0.0001]	2.0316

**Table 3:** Representative physiological parameters of patients at varying pain intensities.

Patients	Pain Intensity	Mean BPM	Mean RR Intervals (s)	Mean Oxygen Level (%)
P1	No pain	71.5895	0.8092	96.2173
	5	74.75	0.8844	96.4166
	8	70.6428	0.7961	95.5
P2	No pain	71.7199	0.8187	96.1968
	3	74.2	0.8154	97
	5	70.24	0.8168	96.52
	8	70.2857	0.7152	96
P3	No pain	84.3372	0.7001	98.4497
	4	82	0.6726	97
	5	84	0.6898	99
	6	84	0.6894	99

Note: P1–P3 are representative participants selected to illustrate heterogeneous response patterns; BPM = beats per minute; RR = R-R intervals.

**Table 4:** Summary of regression model performance.

Model	Variant	R^2^	MAE	RMSE	Note
Linear Regression	-	0.1	3.4	-	Baseline; linear assumption violated
Random Forest	D0	0.088	0.298	0.984	Zero-imputed; biased distribution
	Di	0.261	1.027	1.463	Interpolated; best temporal continuity
	De	0.432	0.929	1.397	Deleted NaN; n = 533 complete cases
ANN	Di	0.23	-	1.494	Non-linear; limited by pain subjectivity

Note: ANN values are reported as averages across three normalisation variants (N1/N2/N3);D0 = zero imputation; Di = linear interpolation; De = deletion of incomplete records. (-) = metric not applicable.

**Table 5:** Classification model performance: precision, recall and F1-score per pain class.

Model	Variant	Class	Prec.	Recall	F1	Acc.	Note
RF	D0	Low	0.978	0.997	0.987	97.46%	Strong bias toward Low class
		Moderate	0.471	0.145	0.222		
		High	0.750	0.125	0.214		High and Moderate severely underdetected
RF	Di	Low	0.470	0.414	0.440	60.65%	Bias toward Moderate
		Moderate	0.666	0.755	0.708		
		High	0.591	0.508	0.546		Most balanced among Di variants
RF	De	Low	0.711	0.771	0.740	76.64%	Most balanced overall; n = 107 test
		Moderate	0.780	0.886	0.830		
		High	0.842	0.571	0.681		Small test set limits confidence
ANN	Di	Low	0.64	0.556	0.595	57.29%	One-vs-rest: Low 70.96%, Mod 64.67%, High 78.79%
		Moderate	0.515	0.66	0.579		
		High	0.53	0.412	0.464		Class-level F1 not reported; see Figure 3

Note: D0 = zero imputation; Di = linear interpolation; De = deletion of incomplete records. RF = Random Forest; ANN = Artificial Neural Network.

## Data Availability

The original contributions presented in this study are included in the article. Further inquiries can be directed to the corresponding author.

## Abbreviations

The following abbreviations are used in this manuscript:

AI	Artificial Intelligence
ANN	Artificial Neural Network
AUC	Area Under the Curve
BPM	Beats Per Minute (Heart Rate)
BR	Breath Rate
BVP	Blood Volume Pulse
D0	Zero-imputed data variant
De	Deleted data variant (listwise deletion)
Di	Interpolated data variant
ECG	Electrocardiogram/Electrocardiography
EEG	Electroencephalography
EMG	Electromyography
F1	F1-Score
FN	False Negative
FP	False Positive
GSR	Galvanic Skin Response
HR	Heart Rate
HRV	Heart Rate Variability
IoT	Internet of Things
LDA	Linear Discriminant Analysis
LR	Linear Regression
MAE	Mean Absolute Error
ML	Machine Learning
NRS	Numerical Rating Scale
PPG	Photoplethysmography
R^2^	Coefficient of Determination
ReLU	Rectified Linear Unit
RF	Random Forest
RMSE	Root Mean Square Error
RR	RR intervals (interbeat intervals)
SCL	Skin Conductance Level
SpO_2_	Peripheral Oxygen Saturation
SVM	Support Vector Machine
TN	True Negative
TP	True Positive
